# Influence of occupant presence patterns on energy consumption and its relation to comfort: a case study based on sensor and crowd-sensed data

**DOI:** 10.1186/s13705-022-00336-6

**Published:** 2022-02-21

**Authors:** Robert Rusek, Joaquim Melendez Frigola, Joan Colomer Llinas

**Affiliations:** Institute of Informatics and Applications, University of Girona, Av. LluisSantaló S/N,Bloc P IV, Research group eXiT, 17003 Girona, Spain

**Keywords:** Crowd-sensing, Energy behavior, Energy efficiency, Indoor comfort, Occupancy patterns

## Abstract

**Background:**

In recent years, the monitoring of occupant presence patterns has become an imperative for building energy optimization. Very often, there is a significant discrepancy between the building energy performance predicted at the design stage and the actual performance rendered during the building operation. This stems from the difference in user occupancy. In spite of this, user interaction and feedback are rarely taken into account and evidence of the impact of occupant presence patterns on energy consumption is still scarce. Thus, the purpose of this study is to apply crowd-sensing techniques to understand how energy is consumed and how appropriate performance indicators should be defined to provide inputs for building operations regarding more efficient use of resources.

**Methods:**

Monitoring strategies were implemented in an office lab with controlled variables to collect quantitative data on occupancy patterns, ambient factors and energy consumption. In addition, crowd-sensing techniques were applied to model user activity in different ambient conditions over time and to contrast their occupancy with energy consumption patterns in combination with new inquiry tools to identify how occupants perceive their comfort level. In addition, a set of energy efficiency indicators was used to compare energy performance over different periods.

**Results:**

It was discovered that there is a strong relation between user occupancy patterns and energy consumption. However, more than 50% of energy was consumed when no user activity was registered. Energy performance indicators revealed that measuring energy efficiency in terms of kWh per surface area encourages a less efficient use of space and, therefore, including a coefficient of person hours is advisable. It was also discovered that users do not fully rely on feedback mechanisms and they prefer to take action to adapt the ambient conditions rather than simply expressing their opinion. Analysis of energy usage during the Covid-19 lock down revealed substantial use of energy contrary to what was expected. This was because home computers were used as terminals only, while the actual tasks were performed on the lab computers, using remote desktop connections, which were turned on 24/7. In addition, energy consumed by each employee at his/her home should be taken into account. Moreover, a set of practical recommendations was formulated.

## Background

In recent years, monitoring of user occupancy has become an imperative for building energy^1^ optimization, as it was realized that buildings do not consume energy by themselves but rather it is their occupants who create the energy demand and expect it to be satisfied. It is widely accepted that there is very often a significant discrepancy between building energy performance predicted at the design stage and the actual performance delivered when the building is in operation due to the difference in user occupancy [1–3]. In the design phase, the operational energy consumption is typically simulated using standard occupancy schedules [4]. Such predictions may differ by 46% from the real energy demand [5]. What is more, lack of proper adaptation of building management based on user occupancy may lead to a 30–50% waste due to misuse and non-optimal management [6]. In addition, there is a mismatch between initially designed and real building uses (activities, occupancy, etc.) which evolve continuously. Changes in building experience over time are refurbished or repurposed, used in different ways or host completely different activities than those they were designed for. These circumstances have direct implications on building performance and demand new strategies for better user occupancy and energy demand sensing to perform in an optimal way.

### Influence of user occupancy patterns on energy consumption

Occupancy pattern reflects the way a building (or a part of a building) is used. It allows to see the relationship between space, the number of users and the time they spend in it. It is an important part of user behavior which additionally includes occupants’ interactions with operable windows, lights, blinds, thermostats, and all appliances that allow to adjust indoor conditions [3]. The importance of user occupancy patterns for energy efficiency has been stressed in numerous studies for over a decade, e.g., in [7–14]. In spite of this, in real world cases, occupancy of tertiary buildings is rarely monitored in a reliable way, and the estimations often rely only on the observations of facility managers or surveys that may present inaccurate results [15]. Ericson et al. [16] demonstrated the potential for 42% energy savings using real time occupancy data based on a sensor network, and Taylor [17] has shown over 10% energy cost savings in a large-scale sample of 280 buildings.

The COVID-19 pandemic showed very clearly the impact of occupancy on energy consumption. Previously, there was a strong emphasis on promoting interaction between co-workers which was reflected in the building design [18]. However, the pandemic has changed human behavior and limited interactions drastically. Social distancing and teleworking have had a substantial impact on working conditions and significantly altered energy consumption patterns.

### Energy consumption and user comfort balance

Another challenge is how to achieve a balance between the optimal use of energy and comfortable conditions for the users. Since people spend a large part of their life indoors, the sensation of comfort or discomfort can have a great impact not only on their productivity, but also on their health and general well-being [19]. This is why user comfort should always be taken into account as an integral part of energy management strategies [20]. For this reason, various indoor comfort standards were developed, such as the American Standard ASHRAEE 55 [21], or European ISO Standard 7730 [22], both aimed at defining the optimal indoor comfort level for working conditions. Despite the validity of these standards, gaps between the defined comfort conditions and those experienced by occupants have been perceived [1]. People from different geographical locations have shown differing comfort expectations depending on where they come from [1]. Other factors, such as differences between genders may also play a role [23]. Moreover, controls of heating, air conditioning, window opening or even light are often inaccessible to users due to a lack of will or for safety reasons, leaving individual preferences and comfort needs unaddressed. This is why the opinions of users should be considered as complementary to comfort standards to efficiently manage energy usage, on the one hand, and ensure comfortable indoor conditions, on the other.

### Acquisition of energy-related user behavior data

Understanding the way in which energy is consumed, requires awareness of user activity and space occupancy. A variety of methods which rely on human researchers, such as surveys, focus groups or human observation, have been used in behavioural studies for this purpose [24]. However, collecting accurate, longitudinal data on user presence patterns still remains a challenge.

Historically, building occupants have been underutilized as a source of information, while the key to ensuring a balance between energy efficiency and comfort lies in data on user occupancy in conjunction with their feedback and opinion [25]. However, recently many alternative approaches became available to deal with this issue. Salimi and Hammad [26], based on a profound literature review, distinguished the following types of occupancy monitoring techniques: (1) Motion sensors that are used to determine the occupant presence in a space without determining their location; (2) Vision-based localization technologies that take advantage of static cameras to acquire information regarding occupant presence, location, number, and type of activity; (3) Radio frequency-based localization technologies that allow for detection and positioning of an object in a space; (4) Multi-sensor networks that combine different monitoring technologies, e.g., motion sensors and CO_2_ sensors; and (5) Surveys and in-person observation which are also used for collecting occupancy information either combined with other tracking technologies or alone. Moreover, virtual occupancy sensors are an alternative to special purpose occupancy sensors that use desktop activity or energy meters to provide indication of occupants’ presence without having to install special purpose occupancy sensors.

Independently, Rueda, et al. [27] identified the following occupancy detection methods: (1) passive infrared sensors to detect motion; (2) environmental sensors, e.g., CO_2_; (3) smart meters that reveal occupancy based on energy consumption; (4) Wi-Fi and Bluetooth that detect the presence of smart devices, and (5) sensor fusion—a mixture of the above-mentioned approaches.

Also, regarding the degree of granularity, Salimi and Hammad [26] distinguished four levels of occupancy modelling: The first one is a monitoring at the building level to reveal the number of occupants. The second one shows the state (occupied/unoccupied) of specific spaces within a building. The third one, considers the number of occupants in specific building spaces; and the fourth, occupancy modelling at the occupant level, which provides information on activity of each individual.

This study uses a crowd-sensing approach that takes advantage of Wi-Fi connectivity combined with motion sensors, smart meters and in-person observation for validation purposes. In addition, environmental sensors and surveys are used to collect data on user’s comfort. Regarding the levels of occupancy monitoring, this study falls within the fourth category, since it evaluates activity of each occupant.

Crowd-sensing is an emerging paradigm that empowers users to provide data sensed or generated from their mobile devices (telephones, wearables, etc.). It exploits the concept of a human sensor to provide an insight on occupancy patterns, improving observability of spaces and the way users interact with the indoor environment [28]. Since energy consumption in buildings depends in a large part on user activity [16], crowd-sensing may support energy management by providing highly valuable information on occupancy and user interaction, but also gives the users the possibility of expressing their needs.

## Methods

This paper examines the relation between users’ indoor activity and energy consumption and provides practical recommendations regarding a more efficient use of resources. It aims to: identify occupancy patterns and find out to what extent these patterns are correlated with energy consumption; verify importance of user activity on energy consumption with a set of performance indicators; examine the possible relation between users’ comfort and energy consumption; and find out how COVID-19-related lock down affected the user behavior and energy consumption.

For this purpose, an experiment was carried out in an office lab to monitor user activity and related energy consumption (objective data), as well as the level of user comfort (subjective data). For comparison purposes, the data was collected in four 4-week periods: spring, summer, autumn and winter. The details of the case study are presented in “Case study description”, “Time span of the case study”, “Participants of the case study”, “Objective vs. subjective measurements” and “Data preparation and preprocessing” sections.

### Case study description

**Fig. 1:**
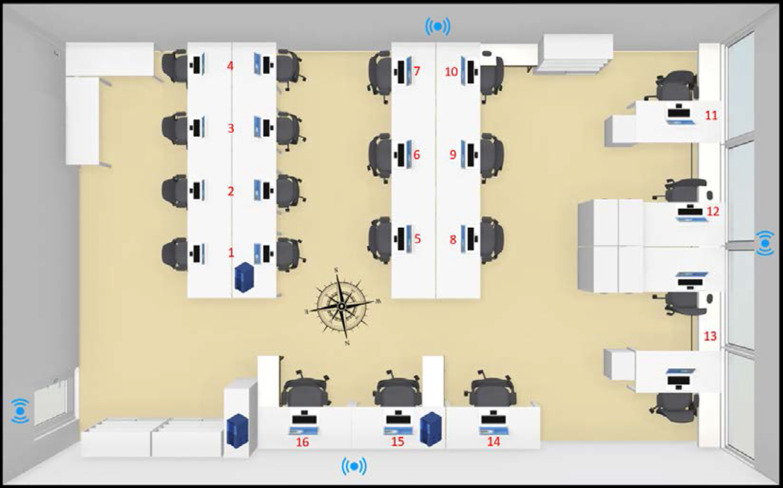
Overview of the 72m^2^ office lab where the experiment took place

Monitoring strategies were implemented in an office lab with controlled variables to facilitate the validation and collection of qualitative data. This aimed to model the user presence and energy consumption patterns in different indoor environmental conditions. The experiment took place in a research facility building located in Girona, Spain, with a Mediterranean climate and with maximum average temperatures of 18.3 °C in February and of 33 °C in July [29]. The office lab, with a surface area of 72 m^2^ and illuminated with fluorescent lighting was chosen to carry out the study. The lab consisted of 16 workstations with desktop computers and had been equipped with sensors measuring environmental indoor conditions. The workstations were numbered 1–16 according to energy supply sockets (workstation 13 is shared) and the location of four sensor kits were marked with blue icons and can be seen in Fig. 1. In addition, each workstation was equipped with sensors measuring energy consumption. The sensors were installed in accordance with the manufacturer's specification to cover the entire floor area. For this purpose, also a series of tests was carried out in which the occupants entered, moved around and left the lab. At the same time, their behavior was observed and compared with the data sent by the sensors in real time. It turned out that there was no case of a place, where the occupant’s activity was not detected, and vice versa, where the presence of a person outside the laboratory was mistakenly considered. Moreover, the order of detection allowed for the identification of the movement of users inside the laboratory. In addition, the sensor data was verified with crowd sensing data from the users’ mobile devices to obtain a reliable occupational pattern.

According to Pérez-Lombard et al. [30], heating and air conditioning accounts for 50% of the energy consumed in office buildings, while the other 50% is due to user-related activities, such as lighting and appliances. This study specifically focuses on energy consumption due to user-related activities. For this reason, the energy consumption of the workstations and lighting were taken into account as these two elements are directly influenced by user activity. Central heating and air conditioning systems were not included in this study as these are centrally managed and their energy consumption does not depend on the number of users. Furthermore, the relation between temperature and energy consumption due to the heating and air conditioning systems is clear and has been the subject of many previous studies [31–33].

### Time span of the case study

Building energy behavior tends to show some typical annual, weekly and daily patterns [34]. Thus, to measure possible differences in occupant presence patterns and energy consumption patterns over different time periods, the experiment was divided into two phases. The first phase covered a 4-week period in July 2019 and a 4-week period in February 2020. Later, in the second phase, the results of the experiment were compared with two other 4-week periods: spring and autumn 2020.

### Participants of the case study

There were 16 users (employees, occupants: 2 females and 14 males) in the summer period and 14 users (2 females and 12 males) in the winter period, as well as 4 users in the autumn period. Due to the lock down caused by the COVID-19 pandemic, user participation in the spring period was not possible. The employees performed their usual daily tasks without being advised that their energy-related behavior was being monitored to prevent the results being altered. However, they were asked to provide feedback on their satisfaction with comfort level and also had the possibility of adjusting some of the factors influencing their comfort (light, access to the thermostat, the possibility of opening windows). An automatic reminder was set up to notify the users about giving their opinion. One of the authors of this article was also present in the case area throughout the whole duration of the experiment.

### Objective vs. subjective measurements

To uncover user presence patterns and their possible relationship with energy consumption and environmental factors, two types of data sets were required: objective (sensor-based) and subjective (user-based).

Objective aspects were strictly related to energy behavior (time and amount of energy used per workstation), environmental indoor measurements (temperature, humidity, pressure, noise level, light, door/window opening) as well as occupancy (time and space). Many studies attempt to increase energy efficiency using occupancy schedules for simplicity and due to a lack of readily available data [12]. In this study, real occupancy data from sensors combined with crowd-sensed data from the users’ mobile devices were used. These data were complemented with external environmental data deriving from the weather station installed outside the lab and energy consumption data. All types of data were stored with a frequency of 10-min intervals.

**Fig. 2:**
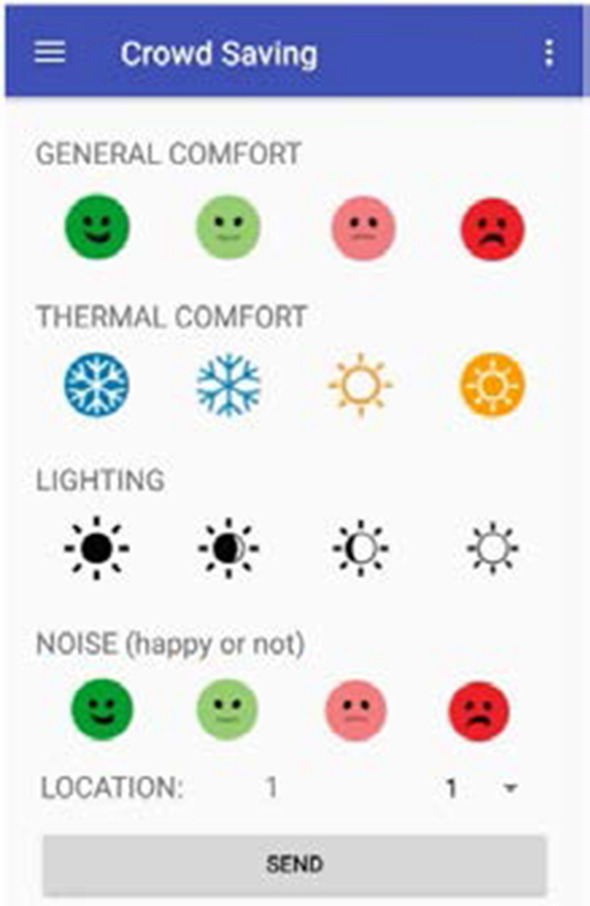
User interface of the app for crowd sensing the occupants behavior and feedback on level of comfort sensation

Subjective aspects were associated with the occupants’ perceived comfort levels. For the collection of the subjective data, crowd-sensing techniques were applied. To uncover the occupancy patterns and find out users’ opinion on comfort levels, a mobile app was developed and the occupants were asked to voluntarily install it on their devices. The app recognized users’ location based on the Wi-Fi signal and gave users the possibility of expressing the level of comfort with regard to four aspects in accordance with the scale for comfort and temperature sensation proposed by Gagge et al. [35]: general comfort (comfortable, slightly uncomfortable, uncomfortable, very uncomfortable); thermal comfort (cold, slightly cool, slightly warm, hot); visual comfort (very dark, slightly dark, slightly bright, very bright) and acoustic comfort (silent, slightly noisy, noisy, very noisy). The app presented this scale in an intuitive and user-friendly manner, as shown in Fig. 2.

It must be stressed that the use of personal devices for data collection of user occupancy patterns may raise concerns regarding privacy violations and security risks. Therefore, for the purpose of this study, the participants were assured that data would be aggregated in order not to reveal personal details.

### Data preparation and preprocessing

Assuring good quality of data is essential for data analytics to obtain reliable results and in consequence, draw accurate conclusions. Therefore, prior data preparation was necessary. For the purpose of this experiment, the following data sets, encompassing 4-week periods each, were obtained:

- July 2019 (summer)—objective, sensor-based data and subjective users opinion;
- February 2020 (winter)—objective, sensor-based data and subjective users opinion;
- April 2020 (spring)—objective, sensor-based data;
- November 2020 (autumn)—objective, sensor-based data.

First, objective and subjective data sets were merged. The objective data were saved with a 10-min frequency; however, the subjective data were saved asynchronously at the time the users provided feedback. For this reason, the time stamp of the subjective data sets was rounded to the nearest 10 min and both data sets were merged. Then, to deal with the missing values in sensor-based data sets, an interpolation for cyclic weekly data was created—a linear interpolation between the last known week, day, hour, and minute with the next known same week, day, hour, and minute. For initial or final missing values, the last available day values were used, and in case of variables, such as temperature, the closest known value was used.

The light consumption data, which presented some error values due to a temporary malfunction of the sensor, were recreated based on the occupancy pattern. Due to the low amount of natural light in the lab, artificial illumination is always used whenever users are present (regardless of the number of occupants); therefore, the curve of energy consumption due to lighting follows the occupancy pattern. Taking this into account, and the fact that energy consumption of lighting is almost binary—0 when it is off and approximately 23 Wh (Light 1) and 56 Wh (Light 2) when it is on—the corrupted data were recreated according to the occupancy pattern.

The fact of performing such data pre-processing allows for further analysis, reliable results to be obtained and, in consequence, well-founded conclusions to be drawn.

## Results

### User occupancy and energy consumption patterns

**Fig. 3:**
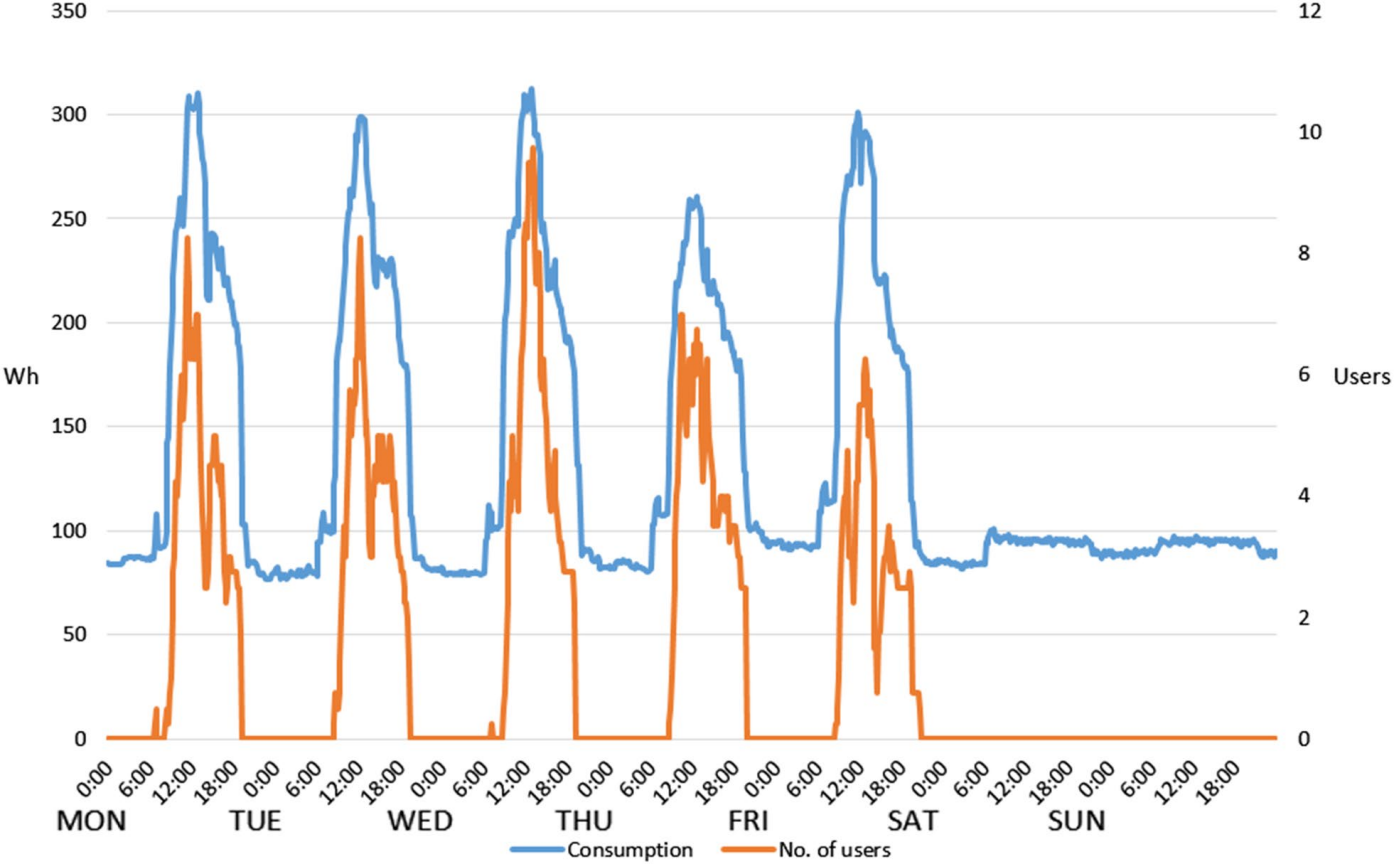
Relation between occupancy pattern (user behavior)

Energy consumption patterns and their relationship with user activity, comfort and environmental factors were investigated to uncover the energy saving potential. Unlike residential buildings, where the behavior of inhabitants is not conditioned by strict time schedules, occupancy of office buildings is usually a result of well-defined timetables. However, the number of occupants varies throughout the day. Thus, occupancy patterns and their relation with energy consumption have been examined in the example of the office lab. As Fig. 3 demonstrates, the number of users increases from around 9:00 am and reaches its peak just before the midday. Then, around 1:00–2:00 pm it decreases as a result of the lunchtime break, and rises slightly again in the early afternoon, going down completely at around 6:00 pm when users leave for home. This pattern has been obtained from averaged values encompassing a period of 4 weeks and has proved to be very similar for all weekdays. In addition, on Monday and Wednesday some minor activity can be observed before 9:00 am due to the cleaning service which maintains the lab twice a week.

A strong correlation between user occupancy patterns and energy consumption can be noticed as both curves are relatively parallel. In addition, the Pearson correlation coefficient was calculated to verify this observation. This indicator calculates the correlation between two variables and returns to a value from −1 to 1, where *−*1 represents a total negative correlation and 1 a total positive correlation, whereas 0 denotes no correlation [36]. The result of 0.93 was obtained, confirming a strong positive correlation.

Nevertheless, a significant use of energy is observed even in periods with no user activity: evenings, nights and weekends. This is due to the consumption from three servers and some workstations executing computation tasks which are turned on constantly. The total energy consumption generated by users of the office lab, during the two 4-week periods—summer 2019 and winter 2020—is summarized in Table 1.

**Table 1:** Comparison of energy consumption in summer and winter periods

	Summer 2019		Winter 2020	
	kWh	%	kWh	%
Total energy consumption	543.2	100	555.2	100
Energy consumption working hours^a^	259.0	48	216.8	39
Energy consumption afterhours^b^	284.2	52	338.3	61

^a^At least one person present

^b^No users present

This clearly demonstrates that in spite of an unquestionable correlation between energy use and occupancy, the majority of energy consumption (52% in the summer period and 61% in the winter period) occurred after hours when no user activity was registered.

**Fig. 4:**
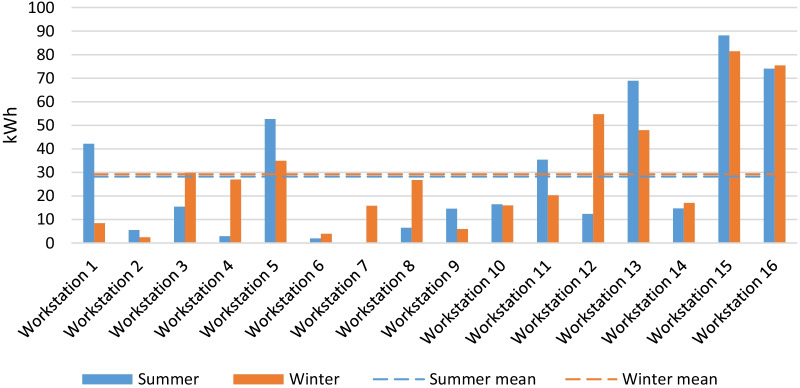
Comparison of energy consumption per workstation in the summer and the winter period

To get a better picture and identify the major sources of consumption, the energy demand of the workstations was compared for the summer and winter periods in Fig. 4.

As Fig. 4 shows, workstations 1, 5, 11, 12, 13, 15 and 16 were the most energy intensive with consumptions exceeding the mean value. The distribution of appliances in the lab shown in Fig. 1 helps to understand this result. In the summer period, workstation 1 was shared with a server, which was later removed. This explains the significantly lower energy consumption in the winter. On workstation 5, a very powerful machine was used to process complex tasks which resulted in higher consumptions than those of the other workstations. Occasionally some other devices were also plugged in, which impacted the total consumption. The same applies to the workstation 12 in the winter period. Workstation 11 was turned on almost all the time in the summer period and turned off for the weekends in the winter, which explains the difference in consumption between these two periods. Number 13 encompasses two workstations located in close proximity, and workstations 15 and 16 were shared with servers. Thus, the major factors that influenced energy consumption were servers and computers which were switched on constantly.

### Energy efficiency indicators

To obtain a deeper insight and to measure energy efficiency of the office lab, a set of indicators was used. The most common one correlates energy consumption with space area (kWh/m^2^); however, it was argued by Huovila et al. [15] that it may be useful at the design and planning stage but it omits the actual user activity which is crucial in the building operation phase. User activity has enormous impact on building energy performance which may lead to a considerable discrepancy between the predicted and actual energy consumption of buildings [37, 38]. For this reason, understanding the human activity and interplay of building occupancy and energy consumption is essential. This is why the kWh/m^2^-indicator was complemented with others that consider the human factor: (1) kWh/person which correlates the use of energy with the number of occupants; (2) kWh/person hours, which correlates the energy consumption with the actual sum of the number of hours that users spend in the building, or the specific area, during the given period of time which for the purposes of this study were periods of 4 weeks; 3) kWh/m^2^ person hours, which combines indicators (1) and (2), to take the floor area into account as well as the occupancy time according to the formula:

*Energy per area per occupied hours* = $\frac{kWh}{{m}^{2} h}=\frac{\frac{kWh}{{m}^{2}}}{h} \times 1000$, where *kWh* is the amount of energy consumed over the given period of time, *m*^*2*^ is the floor area of the space in question, and *h* is the number of hours the space was occupied during the given period [39].

The energy performance of the office lab according to these four indicators is compared in Table 2. In addition, for comparison purposes, the numbers of kWh per workstation and kWh per person were included.

**Table 2:** Comparison of energy indicators of the office lab in the summer and winter periods

	m^2^	No. of users	kWh	m^2^/person	kWh/m^2^	kWh/workstation	kWh/person	kWh/person hours	kWh/ m^2^, person hours
Summer	72	16	543.2	4.5	7.5	28.2	34.0	3.1	43
Winter		14	555.2	5.1	7.7	29.3	39.7	3.9	54

In spite of a greater number of users, perversely less energy was consumed in the summer period. However, the difference of 2.2% is within acceptable limits. The Spanish norm for occupancy defines the standard as a minimum of 5 m^2^ per person for this type of space [40], which means that in the summer period, the office lab was slightly over-occupied with only 4.5 m^2^/person. This translated into higher energy efficiency as shown by other indicators. It is worth noting that the difference between these two periods is particularly noticeable in terms of those indicators that involve a human factor. This is because factors of energy use which are dependent on the number of users are higher, while the floor area of the building remains constant [15].

### COVID-19 lock down and post lock down impact on office energy efficiency

The year 2020 was unprecedented and differed from previous years in many aspects among which energy use and energy related human activity are no exception. In Spain, during the spring and summer of 2020, users carried out all their tasks from home due to the lock down. Then, in the autumn, employees returned to their offices, but only partially, with limited hours and working partially from home. Table 3 shows the energy consumption of the office lab in these two periods.

**Table 3:** Comparison of energy consumption in spring and autumn periods

	Spring 2020		Autumn 2020	
	kWh	%	kWh	%
Total energy consumption	420.4	100	322.7	100
Energy consumption working hours^a^	–	–	72	22
Energy consumption afterhours^b^	–	–	251	78

^a^At least one person present

^b^No users present

It can be seen that in spite of the lack of physical presence of employees in the spring period, energy consumption is significantly higher than in the autumn when employees partially returned to the office. At first glance, this could be interpreted as an inverse relationship between occupancy and energy consumption. However, it is because some employees used home computers as terminals only, while the actual tasks were performed on the lab computers using remote desktop connections. These computers were not turned off every day after work (as normally happens), but were turned on 24/7 instead, which explains higher energy consumption. Since during the autumn period, work was carried out in a semi-remote mode, the energy consumption was lower. However, even still, the greater amount of energy (78%) was consumed after hours. Nevertheless, it is important to stress that for both spring as well as autumn periods, the significantly lower energy consumption in comparison with the summer and winter periods was caused by the fact that energy consumed by the employees at their homes was not considered, although it should be included. However, collecting such data are challenging, and therefore, it may be the subject of a separate study.

To gain a deeper insight and show how the drastic change of user occupant presence patterns (home working) caused by the COVID-19 outbreak impacted energy efficiency, a set of indicators has been calculated for the spring and autumn periods and presented in Table 4.

**Table 4:** Comparison of energy indicators of the office lab in the spring and autumn periods

	m^2^	No. of users	kWh	m^2^/person	kWh/m^2^	kWh/workstation	kWh/person	kWh/person hours	kWh/m^2^, person hours
Spring	72	0	420.4	**–**	5.8	26.3	**–**	**–**	**–**
Autumn		4	322.7	18	4.5	20	80.7	4.0	56

Comparison of values from Table 2 and shows how consideration of occupancy is important for providing a more realistic picture of the situation. The kWh/m^2^ indicator for spring and autumn is clearly lower than in the summer and winter periods which may suggest higher efficiency. However, when looking at the indicators that include human activity, a more realistic overview can be obtained. kWh/person, kWh/person hours, as well as kWh/m^2^/person hours are higher in the autumn than in the summer and winter periods (obtaining these indicators for the spring period was not possible due to the lock down). This is because the more effectively a space is used, the more it consumes in absolute numbers and less regarding the number of users. In addition, the higher the occupancy and space efficiency, the less the building or space tends to appear efficient when the indicator of energy consumption per floor area is used (kWh/m^2^), since a greater number of users produce a higher energy demand, while the floor area remains constant [39, 41].

### User's perception of indoor comfort conditions

**Fig. 5:**
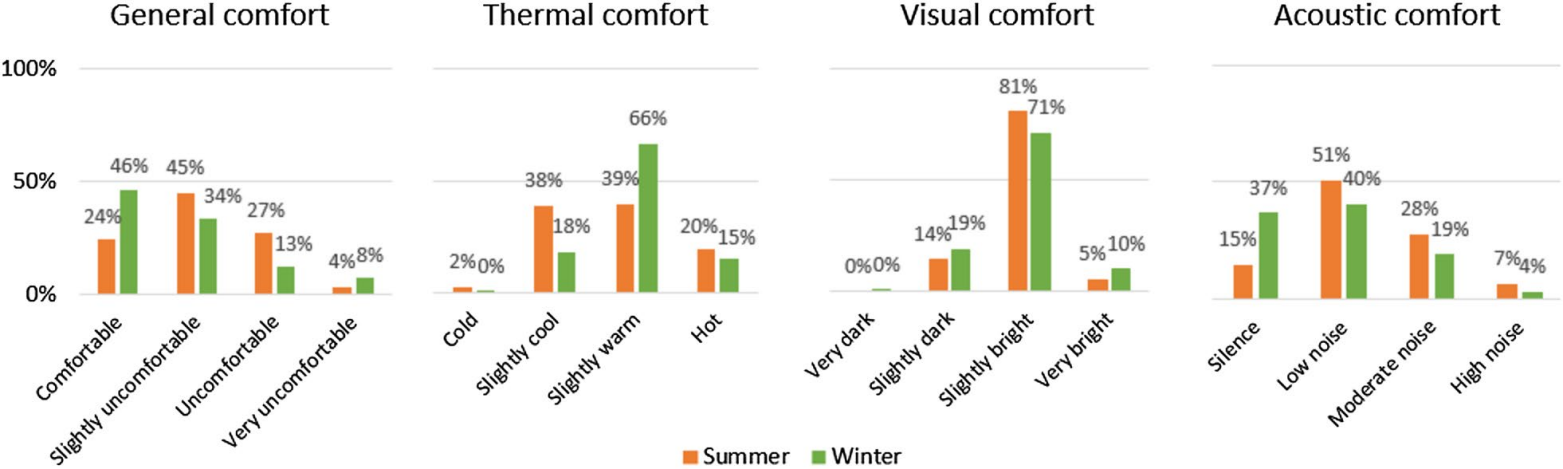
Comparison of comfort levels based on users' feedback for the summer and winter periods

User feedback on comfort was investigated to discover the possible relationship between energy consumption and comfort levels. Comfort can be defined as a condition of mind which expresses satisfaction with the environment [42]. Energy consumption may have a direct impact on such a steady state sensation. For this reason, during the summer and winter periods, users were asked to use a mobile crowd sensing app and provide feedback on their level of comfort. Figure 5 shows the comparison of general, thermal, visual and acoustic comfort levels in the summer and winter (calculated as the proportion of votes for each category over the total number of votes).

**Fig. 6:**
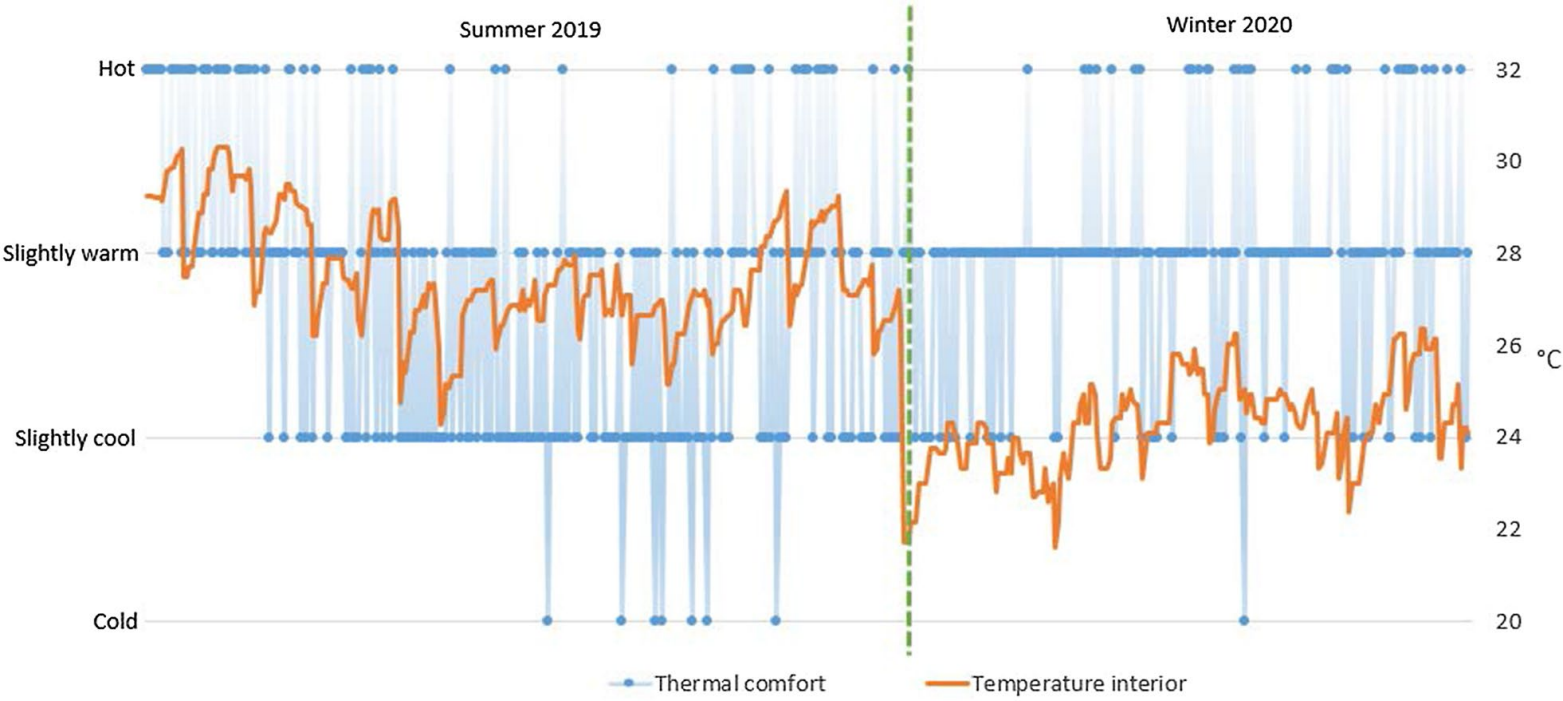
Comparison of interior temperature with thermal comfort in the summer and winter periods

The general comfort in the summer and in the winter was close to optimal with 69% and 80% of opinions being comfortable or slightly uncomfortable, respectively. The result of thermal comfort in the summer period, however, is not so clear with 38% *slightly cool* and only 20% *hot*. Especially surprising is 2% of *cold* in the summer voted when the indoor temperature during working hours fluctuated between 26.7 and 28.7 °C, while the European standard EN 15,251 [43] defines the summer comfort temperature in the range between 23 and 26 °C. Figure 6 shows that there is no strict relationship between temperature and the thermal comfort sensation of users. In particular, it can be seen that the same range of temperatures (25 °C–26 °C) is considered as *slightly cool* or even *cold* in the summer, while it is considered *slightly warm* or even *hot* in the winter. However, for a better understanding of this phenomenon, a separate study is necessary that includes a set of different factors that can influence the feeling of thermal comfort, such as the different clothing level.

**Fig. 7:**
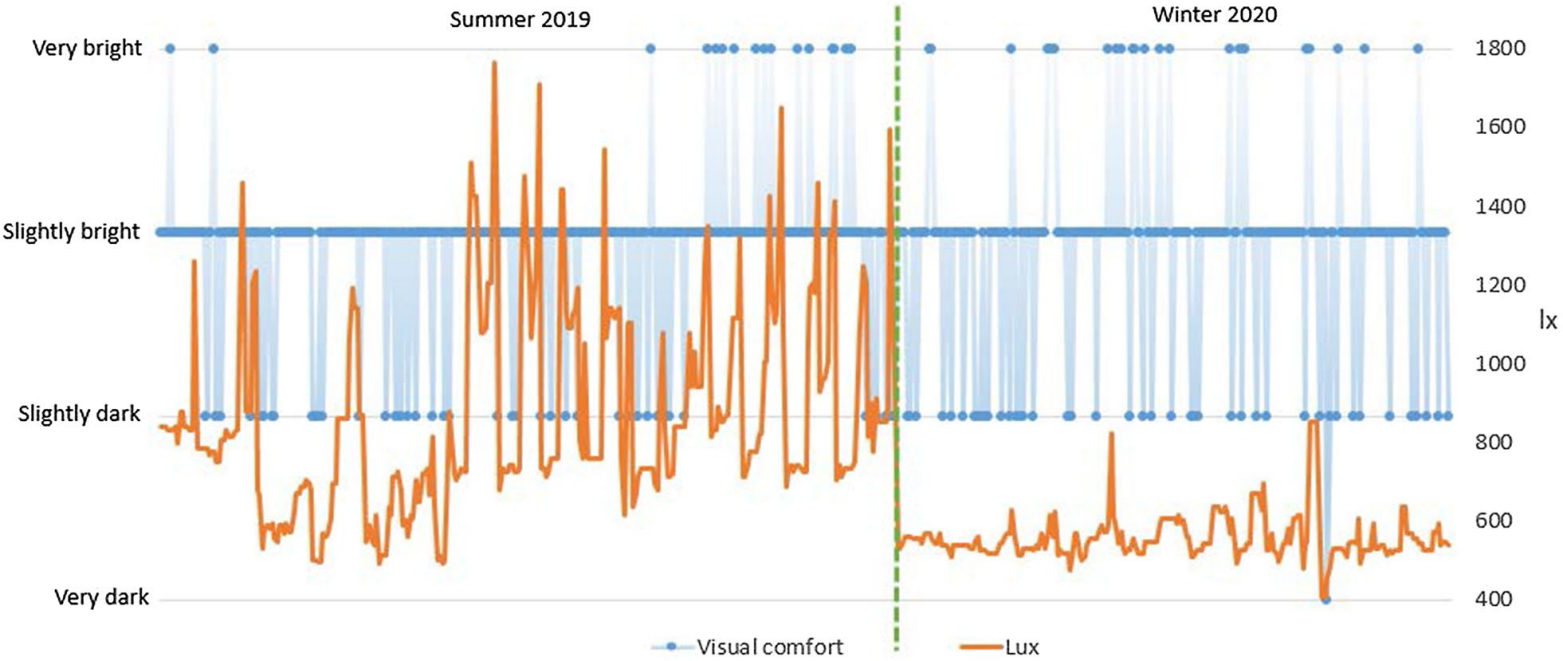
Comparison of light levels with visual comfort in the summer and winter periods

It can be also observed that the lighting conditions were close to optimal (Fig. 7). The greatest number of votes (81% in the summer and 71% in the winter) described visual comfort as *slightly bright*. According to the European standard UNI EN 12464–1 [44], a comfortable minimum illumination level should be between 500 and 700 lx and, indeed, the light intensity in the lab was maintained at the level of > 500 lx almost all the time throughout the experiment. In practice, on many occasions it was much higher, especially in the summer period. What is surprising is that the significantly lower light conditions in the winter period have almost no impact on user satisfaction, which is very comparable for the summer and winter period.

**Fig. 8:**
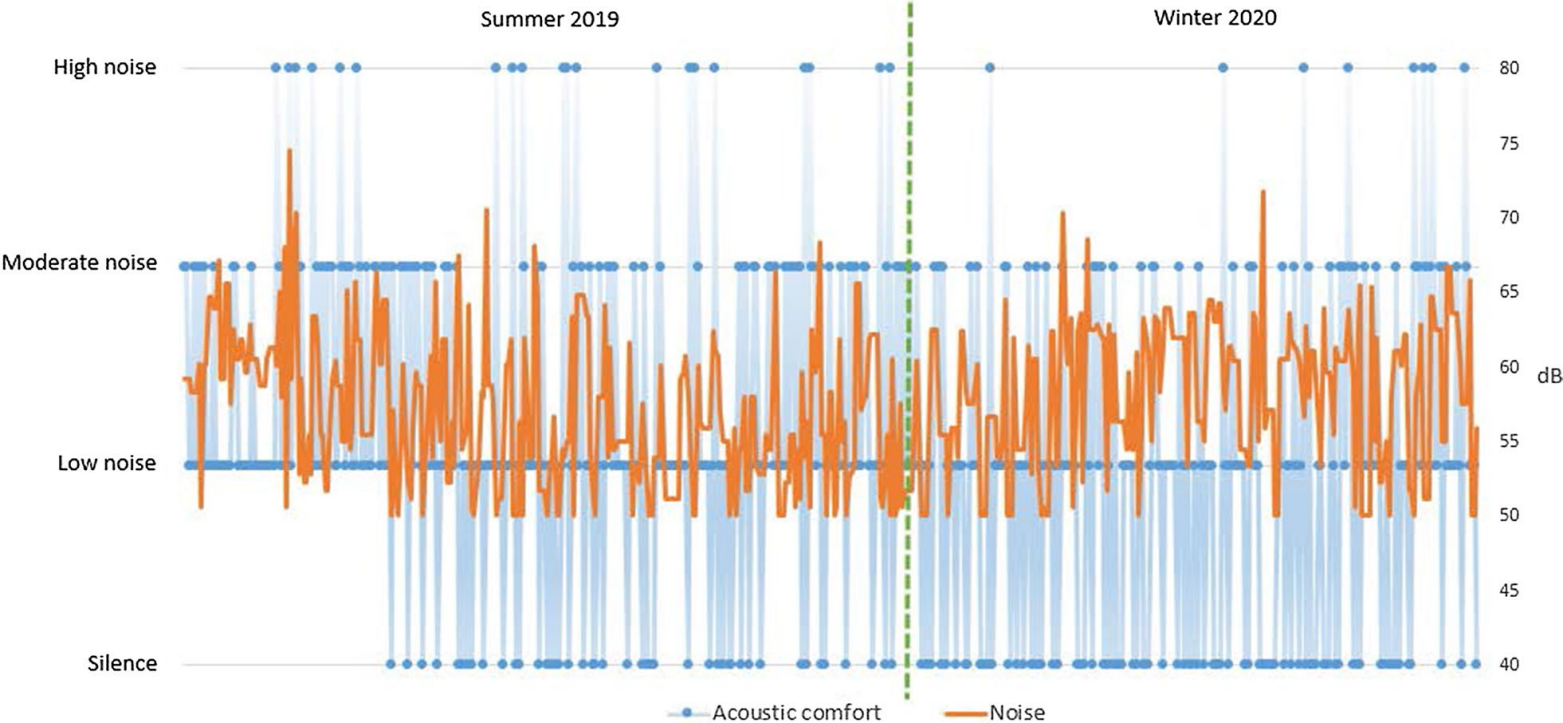
Comparison of noise levels with Acoustic comfort in the summer and winter periods

The noise level was largely satisfactory with a far greater majority of votes for *silence* and *low noise* and with only few complaints about high noise (Fig. 8). The maximum registered values did not exceed the level of 80db which is the limit of comfort defined in the standard (EU Directive 2003/10/EC [45]). Likewise, no significant differences were observed between summer and winter periods. However, it must be stressed that the sensitivity of the sensor was limited and noise levels lower that 50db were not registered.

In terms of gender, the results were almost the same for men and women. However, this result is not objective due to the significant disproportion between the number of representatives of two sexes and no representative of any other gender—16 males and 2 females in the summer period, and 14 males and 2 females in the winter period.

Having said that, it is not surprising that even under optimal conditions that comply with the standard, there will always be some percentage of dissatisfied users. This is because human beings are individuals and there is no unique definition of comfort conditions. Such a thesis was already stated 50 years ago, concluding that there are no ambient conditions that can make all individuals feel equally comfortable [46].

### Level of thermal tolerance

**Fig. 9:**
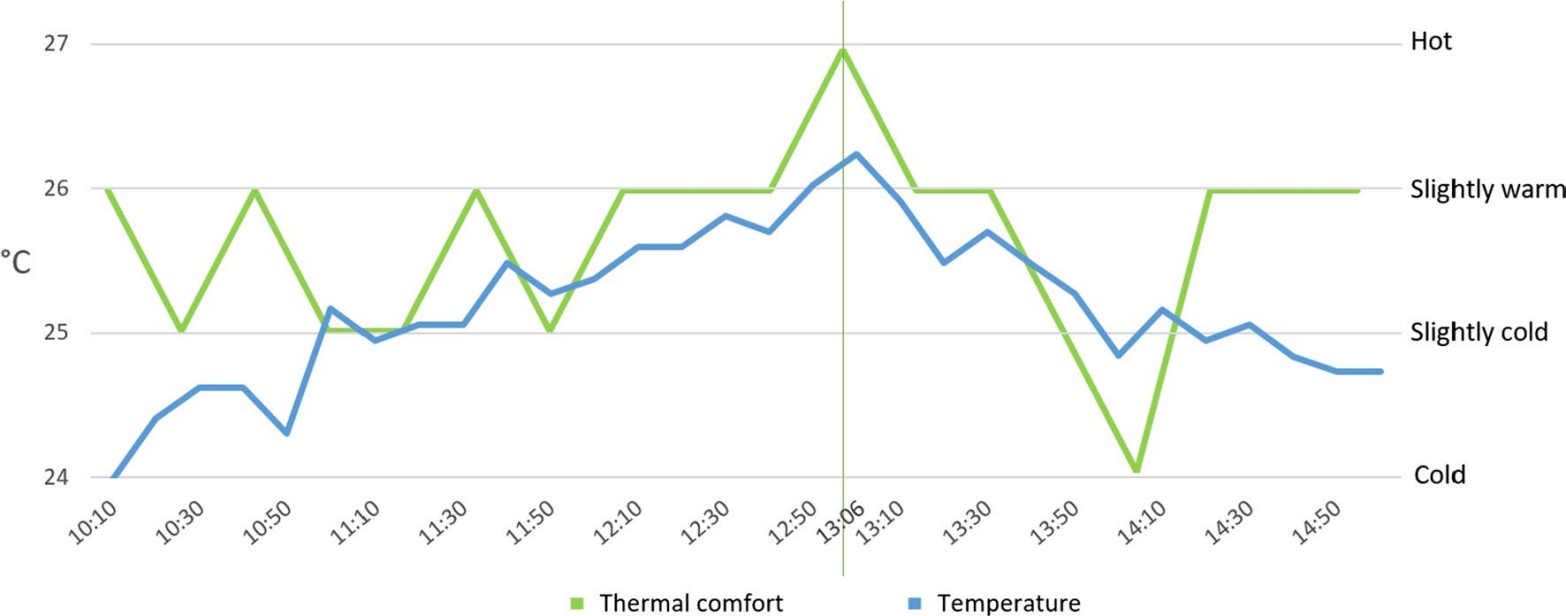
Curve of thermal comfort threshold together with users’ comfort sensation

The comfort or discomfort sensations of users are conditioned by a mixture of different environmental factors, such as humidity and atmospheric pressure, which are not easy to identify unless they reach extreme values. Nevertheless, hot or cold sensation is very easy to pinpoint even if the variation is only of a few degrees. Temperature is the most easily noticeable and most influential factor that determines the level of comfort [47]. For this reason, the level of thermal tolerance of occupants in the office lab was studied. To this end, on February 17, 2020, without notifying the users, the heating temperature was slowly increased to discover the threshold between thermal comfort and discomfort (sensation of heat). It was expected that—at a certain point—users would start to express their dissatisfaction by choosing the *hot* option in the crowd-sensing app. As it can be seen in Fig. 9, this moment was reached at about 13:10, when the temperature exceeded the threshold of 26 °C. After that, a decrease in temperature can be observed until a level of around 24.6 °C was reached, which, in this case, can be understood as the comfort level. This observation was checked and compared with the opinion of users regarding comfort and revealed that, surprisingly, users did not complain much regarding temperature. Only one vote at 13:06 indicates a *hot* sensation. The other responses were more moderate.

Comparing this result with the data from other sensors revealed the cause of the decrease in temperature. At 13:00, all the windows were opened and the heating was turned off. The windows were closed later at 13:50, while the heating remained off for the rest of the day. This demonstrates, that users, in spite of having a possibility to express their opinion on comfort, prefer to act instead. Probably, this is because they cannot see a direct impact of their opinion on the enhancement of comfort. Thus, they prefer to act (turn off the heating, open windows) instead of expressing their opinion, the latter not immediately satisfying their needs.

### Correlation between energy consumption, comfort level and environmental factors

To obtain a deeper insight into energy performance, possible correlations between energy consumption, user comfort and environmental factors were investigated. For this purpose, a Pearson correlation coefficient was used to measure the relationship between each of the individual parameters. First, energy consumption and comfort levels were tested which showed a very weak, almost neutral, correlation with the higher result of −0.2 obtained between energy consumption and thermal comfort. This was an expected outcome given that heating and air conditioning were not considered according to the initial assumptions.

Similarly, no strong correlation between energy consumption and environmental factors was found. The highest result between energy consumption and humidity, equal to 0.4, is too weak to draw any binding conclusions from it.

Also, surprisingly, no correlation between comfort levels and environmental factors was found, although it was expected to reveal a stronger correlation between temperature and thermal comfort, or noise and acoustic comfort, which intuitively seem to be an obvious association. The data, however, reveal no such relationship. The highest result of 0.3 was between thermal comfort and noise. This may indicate that thermal tolerance and the sensitivity towards other environmental factors of the sample group was too diverse to be able to draw binding conclusions.

## Discussion

The analysis of user activity based on sensors and crowd-sensed data uncovered a regular occupancy pattern during the week. Comparison of this pattern with energy consumption revealed a positive correlation between occupancy and energy consumption. In spite of this, it was observed that more than half the energy was consumed after hours when no user activity was registered. This consumption was due to the servers and some workstations being turned on 24/7. To obtain a more in-depth insight, several indicators were used to compare the energy efficiency of the office lab across different periods. This revealed that measuring energy efficiency in terms of kWh per surface area is a useful metric for comparing the physical properties of a building. However, indicators that include a coefficient of person hours are more reliable for measuring performance and, ideally, a combination of both—space and person—hours gives a more objective overview on building operation efficiency.

Analysis of energy usage during the lock down period revealed substantial use of energy when very low or minimal energy usage was expected. This was because some employees used their home computers as terminals only, while the actual tasks were performed on the lab computers with remote desktop connections. These computers were turned on 24/7, which explains higher energy consumption. In addition, a very important aspect to stress regarding home offices is that the energy consumed by each employee at their home was not included. However, it is very important to take this into account as it forms a part of the overall amount of energy consumed. Including this consumption in the amount of energy consumed in the office may reveal that such a combined mode of working is less energy efficient than a fully in-person or fully homeworking mode. Home working produces an extra consumption at the office (workstations turned on 24/7) and at the home of the employee (energy consumed by computers and all the devices needed to fulfill the employee’s duties, as well as indirect consumption which includes heating, air conditioning, light etc.,). However, more research is required to verify this hypothesis.

The comfort level based on the opinions of users revealed a general satisfaction with a relatively small number of extreme opinions. However, further investigation showed that user opinion did not correlate with objective values from sensors and those defined in the standards, especially regarding temperature. Users acknowledged feeling comfortable, while the temperature values were beyond the range defined in the standard. A further experiment showed that users do not rely on the comfort feedback mechanism, probably because they cannot see a direct impact between expressing their opinion and an immediate adjustment in the ambient conditions. They prefer to act and adjust the environmental parameters by themselves (turning off the heating, opening windows, etc.) rather than voting. Comfort standards are general, but the actual comfort or discomfort sensation is very personal and related to such aspects as a user’s gender or provenience. This is a very important factor to bear in mind as building managers usually adjust ambient conditions according to the standards. This study shows that objective conditions defined in standards may diverge from those perceived by the users. For this reason, a more user-centered approach in building operations should be sought.

To discover a possible correlation between energy consumption, comfort, and indoor ambient factors, a Pearson coefficient was calculated. This exercise, however, showed very weak, close to neutral correlations which were not sufficient to be able to draw binding conclusions.

## Conclusions

Based on the results of this study it is possible to define a set of actions whose purpose is to reduce energy consumption. First of all, the space was occupied unevenly, leading to high concentrations of users during short periods of time and leaving the space unoccupied for the majority of the time. Changing the distribution of users or adjusting the working hours to increase occupation would lead to a more efficient use of space and energy.

Servers contributed considerably to the overall consumption. Since they form an important part of the tasks performed in the lab, they cannot be turned off. Nevertheless, there are still some possibilities to reduce their impact. The servers may be replaced by newer and more efficient devices. Migration to one physical machine with fewer virtual servers should also be considered. It was calculated that reducing the number of servers to one by creating virtual machines would decrease the energy consumption by 14.5% in the summer and by 12.3% in the winter period, which is a considerable amount.

It was also observed that some workstations were turned on constantly, this being the cause of the majority of energy consumed. It should be further analyzed whether there is a need to keep these computers on all the time or to see whether they could be turned off at least for some periods. An ideal, hypothetical scenario in which all workstations were turned off when unused, would allow for a reduction in energy consumption of 26% in the summer period and 36.5% in the winter period. This shows that there are extensive opportunities for energy optimization. The combination of operational efficiency (optimization of servers) together with behavioral changes of users (turning off the workstations when unused) offers great opportunities for optimization in the range of 40.5% in the summer and 48.8% in the winter period, which is in line with the 30–50% as shown by Lasla et al. [6] and 42% energy savings demonstrated by Ericson et al. [16].

This is strongly related to energy education and energy awareness which should go on hand-in-hand with operational energy efficiency. Users should be informed of the amount of energy they consume and its impact in terms of the economic and environmental footprint. An eco-feedback mechanism could be used for that purpose. It is an effective tool that provides the occupants of a building with information regarding their energy consumption, usually in a user-friendly way. Scientific energy units are not easy for everyone to comprehend. However, the information may be presented in a more intuitive form, for example, by the number of trees needed to off-set the carbon footprint produced. Such a presentation of energy impact could offer a good alternative to formal units as users need to understand the potential consequences of their overconsumption. The same mechanism might also advise users regarding their activities and possible changes in their behavior to increase energy efficiency. In addition, the comparison of their consumption to that of their peers is also an efficient tool.

## Data Availability

The data sets used and analyzed in the current study are available from the corresponding author on reasonable request.

## Footnotes

1 This article concerns only electrical energy. Therefore, within the context of this article, energy should be understood as electrical energy.
